# Dynamic distribution and correlation analysis of the angle kappa in myopia patients undergoing femtosecond-assisted laser in situ keratomileusis

**DOI:** 10.1097/MD.0000000000029425

**Published:** 2022-06-17

**Authors:** Wen-Qing Deng, Yu-Hui Fang, Shu-Hua Lin, Ying-Jun Li

**Affiliations:** a Department of Ophthalmology, Fuyang People's Hospital of Anhui Medical University, Fuyang, Anhui, China; b Department of Dermatology, Fuyang People's Hospital of Anhui Medical University, Fuyang, Anhui, China; c Department of Ophthalmology, Affiliated Hospital of Yanbian University, Yanji, Jilin, China.

**Keywords:** ablation centration, angle kappa, coaxially sighted corneal light reflex, pupil centre

## Abstract

**Purpose:**

To explore the offset distribution of pupillary centres, the offset between the pupil centre and the coaxially sighted corneal light reflex (*P-Dist*) and their correlation in femtosecond laser combined with excimer laser in situ keratomileusis.

**Methods:**

Randomly selected 194 patients (398 eyes) who underwent femtosecond-assisted laser in situ keratomileusis with preoperative use of WaveLight Allegro Topolyzer Corneal Topography (WaveLight Laser Technologies AG, Erlangen, Germany) to measure the pupil size and centre position. The *P-Dist* of the patients was recorded by the *X* and *Y* axis eyeball tracking adjustment program of the WaveLight Eagle Vision EX500 excimer laser system.

**Results:**

The *P-Dist* was 0.214 ± 0.092 mm in the right eyes and 0.228 ± 0.105 mm in the left eyes (*P* = .041). Under scotopic conditions, the pupil centre of left eye *X*-axis was −0.046 ± 0.091 mm, the right eye was −0.152 ± 0.084 mm, with significant differences (*P* = .015), and the *Y*-axis direction showed no significant changes (*P* = .062). The white to white was positively correlated with changes of pupil diameter (scotopic pupil diameter-photopic pupil diameter) (*r* = 0.270, *P *< .001). The equivalent spherical mirror and measured centroid shift were negatively correlated (*r* = −0.214, *P* = .002).

**Conclusion:**

The angle kappa of the right eye is smaller than that of the left eye and from scotopic to photopic condition, the pupil centroid shift of both eyes to the nasal inferior side. If the cornea is too large, the low illumination environment should be maintained during the operation to improve the efficiency of pupil matching.

## 1 Introduction

The ideal excimer laser ablation centration should be completely overlapped with the visual axis in corneal refractive surgery.^[1]^ If the pupil positioning tracking scan does not consider the adjustment of the angle kappa, the actual corneal ablation centration will not be consistent with the ideal ablation centration, which will cause “surgical origin” eccentric ablation. Therefore, it is particularly important to adjust the angle kappa during surgery.^[2]^ However, the visual axis is so difficult to establish that the eye tracking system usually locates and tracks the centre of the pupil in actual surgery. Because the pupil centre is different from the visual axis, there is consensus among corneal refractive surgeons that the laser ablation centration from the pupil centre to the visual axis should be adjusted to compensate for the offset effect of angle kappa and to reduce higher-order aberrations after surgery.^[3,4]^

The coaxially sighted corneal light reflex point is the corneal entry point of the visual axis.^[5]^ Studies have shown that the coaxially sighted corneal light reflex point is the ideal ablation centration point because the coaxially sighted corneal light reflex point is the closest point to the visual axis and it is not affected by changes of pupil size or centre position, with an average of 0.02 mm.^[6]^ Therefore, the angle kappa can be understood as the offset between the pupil centre and the coaxially sighted corneal light reflex (*P-Dist*).^[7]^ Nevertheless, the angle kappa is not a fixed value, and it changes with the dynamic change of the pupil centre position.^[8,9]^ That is why it is necessary to further explore the dynamic changes of the pupil size and centre position suitable for excimer laser surgery, *P-Dist* and its correlation relative parameters.

This study analyses the *P-Dist* and includes correlation analysis to explore the dynamic changes of pupil size and centre position suitable for excimer laser surgery. It provides a reference for the design of surgical solutions as to how to optimize femtosecond-assisted laser in situ keratomileusis with angle kappa compensation in accordance with the optical characteristics of individual human eyes.

## 2 Methods

We included randomly selected 194 (398 eyes) myopia patients who underwent femtosecond-assisted laser in situ keratomileusis at the Ophthalmology Department of the Affiliated Hospital of Yanbian University from January to November in 2019; patients were aged 18 to 40 years.

The inclusion criteria were as follows: (1) Preoperative central corneal thickness ≥ 480 μm, residual corneal stromal bed thickness ≥280 μm; (2) preoperative spherical lenses (−0.50 to −9.50 D), cylindrical lenses (0 to 1.50 D), anisometropia ≤1.50 D; (3) corneal contact lens discontinued >2 weeks; and (4) no recent history of taking medications (glucocorticoids, contraceptives, etc). The exclusion criteria were as follow: (1) corneal diseases, keratoconus, cataract, glaucoma, uveitis, retinal diseases or other eye diseases; (2) diabetes, hypertension, rheumatism, hyperthyroidism or other systemic diseases; and pregnancy or lactation. (3) Informed consent was obtained from all subjects using a consent form approved by the Institutional Review Board of the Affiliated Hospital of Yanbian University (No. 2020131), and all procedures conformed to the guidelines of the Declaration of Helsinki.

All patients were examined for uncorrected visual acuity and best corrected visual acuity, slit lamp examination, comprehensive optometry analysis, corneal thickness, axial length, and anterior chamber depth (ACD), which were measured using the IOL Master (Carl Zeiss Meditec, Jena, Germany); fundus examination and the size and central position of pupil were measured by a WaveLight Allegro Topolyzer Corneal Topographic system (WaveLight Laser Technologies AG, Erlangen, Germany) before operation.

**Figure 1:**
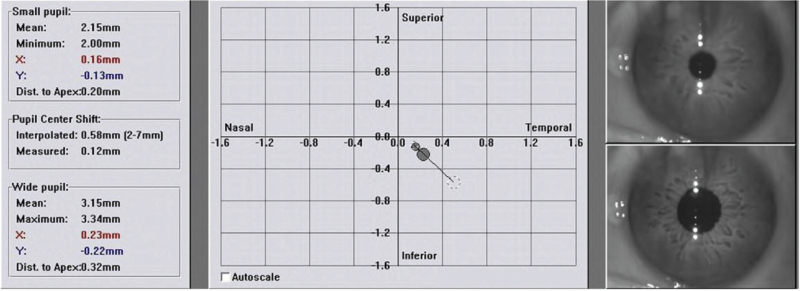
Shown in the table at the left of each image are the data used in the study, the pupil centre in relation to cornea centre (geometric centre of the actual cornea) in the Cartesian coordinates plot, and the actual photopic and mesopic pupil images. In the Cartesian plot, the small circle corresponds to the small pupil apex coordinates, while the large circle corresponds to the large pupil.

**Figure 2:**
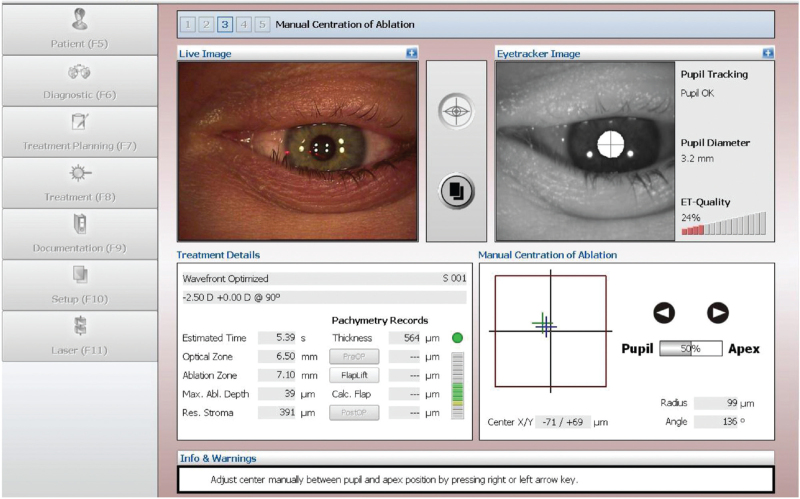
The Corneal light reflex guided centration – offset parameter. It can be determined on the linear distance between pupil centre and corneal light reflex; *P-Dist* (the offset between the pupil centre and the coaxially sighted corneal light reflex).

A WaveLight Allegro Topolyzer Corneal Topographic system measures pupil size and central position under photopic and scotopic conditions. All pupils of subjects were measured and recorded by the same physician under dark room conditions for 60 seconds. A corneal flap with a diameter of 8.5 mm and thickness of 110 mm was created with a superior hinge using a WaveLight FS 200 Hz femtosecond laser (WaveLight, GmbH). After making a flap with a femtosecond laser, we asked the patients to lie flat, and focus on the upper green indicator light. The performer could see the reflective point of the corneal vertex (coaxial corneal reflection point) and the red reflection in the centre of the pupil (optical axis centre, origin of Cartesian coordinate system) under the microscope, and the illumination of the operating microscope and the indoor lighting was adjusted to keep the pupil size consistent with the preoperative examination. If the actual pupil in the treatment image differed in diameter of more than 20% to the diagnostic image, it was possible to modify the actual pupil size and diameter by changing the light conditions using the ‘Microscope/OP Field Illumination Brightness Knob’. The *X* and *Y* axis eye-tracking adjustment program of WaveLight EX500 excimer laser (WaveLight GmbH, Germany) was used to record *P-Dist* (offset between the pupil centre and the coaxially sighted corneal light reflex) while the patient was supine (Fig. 1). The *X* and *Y* axis eye-tracking adjustment program of EX500 excimer laser system was used to record *P-Dist* while the patient was supine. We manually entered 100% *P-Dist* adjustment into the excimer laser device. We moved the excimer laser ablation centre from the pupil centre to the direction of the visual axis (coaxially sighted corneal light reflex) (Fig. 2). Optical zone of 6.5 mm with a blend zone to 9 mm for excimer laser ablation.

### 2.1 Statistical Methods

All statistical analyses were performed using *SPSS software* (*version 19, SPSS, Inc.*). Continuous data were reported as the mean ± SD, and paired and unpaired t tests were used to evaluate the differences. Categorical data were reported in frequencies and percentages, and the chi-square test for the linear trend was used. The relative parameters correlation was analysed by Pearson correlation. A *P* value of less than .05 was considered statistically significant.

## 3 Results

We randomly selected 194 patients (398 eyes) who underwent examinations at Yanbian University Hospital. There were 102 men (52.6%) and 92 women (47.4%), with an average age of 24.58 ± 6.84 years. Ocular dominance occurred predominantly in the right eye (right vs left: (145) 74.74% vs (49) 25.26%; *P* < .001). The average corneal curvature was 42.62 ± 1.23 D (range: 39.32 to 48.91 D); the average spherical equivalent was −4.62 ± 1.24 D (range: −0.50 to −9.50 D); the average corneal astigmatism was −0.79 ± 0.81 D (range: 0 to −1.50 D); and the average eye axis was 26.51 ± 1.49 mm (range: 23.09 to 30.85 mm) (Table 1).

**Table 1:** General information of patients.

	Mean ± SD	Range
Age (yr)	24.58 ± 6.84	18 to 45
SE (D)	−4.62 ± 1.24	−0.50 to −9.50
Sphere (D)	−5.74 ± 1.91	0 to −10.00
Cylinder (D)	−0.79 ± 0.81	0 to −1.50
Corneal *K*-value (D)	42.62 ± 1.23	39.32 to 48.91
Corneal thickness (μm)	526.72 ± 33.26	496.00 to 646.00
IOP (mmHg)	13.60 ± 1.32	12.4 to 20.5
Anterior chamber depth (mm)	3.56 ± 0.51	1.56 to 4.50
Pupil diameter (mm)		
Photopic	3.29 ± 0.49	2.25 to 5.48
Mesopic	6.09 ± 0.74	3.71 to 7.99
Axial length (mm)	26.51 ± 1.49	23.09 to 30.85

IOP = intraocular pressure, SE = equivalent spherical mirror.

**Figure 3:**
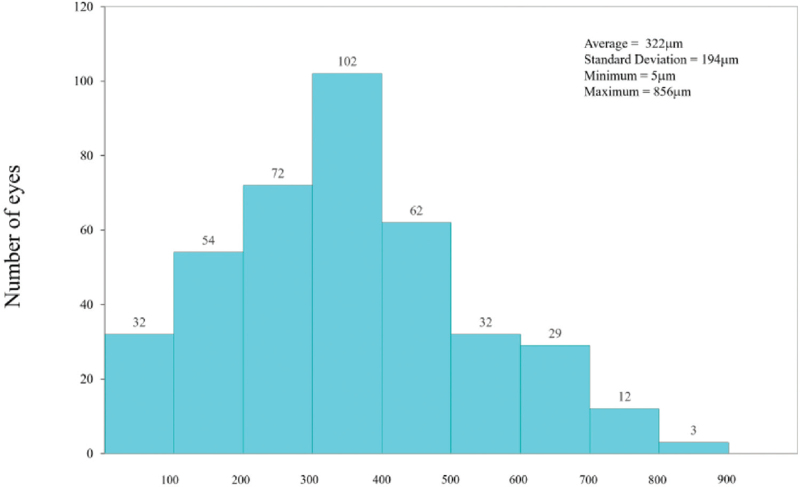
Offset distribution between corneal centre and pupil centre under photopic suitable for corneal refractive surgery patients.

Figure 3 shows the average distribution of the eccentricity between the corneal centre and the pupil centre under photopic conditions, which was 0.322 ± 0.194 mm (range: 0.005–0.856 mm); 21.6% of the eyes were ≤200 μm, 65.3% ≤ 400 μm, 88.9% ≤ 600 μm, and 100% ≤ 900 μm.

**Figure 4:**
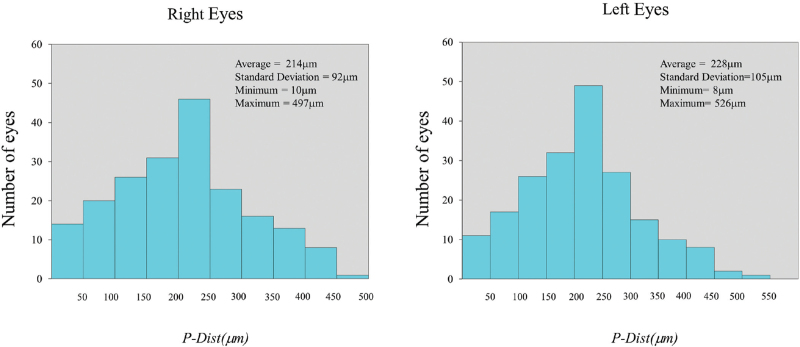
The distribution of *P-Dist* in patients with corneal refractive surgery.

**Figure 5:**
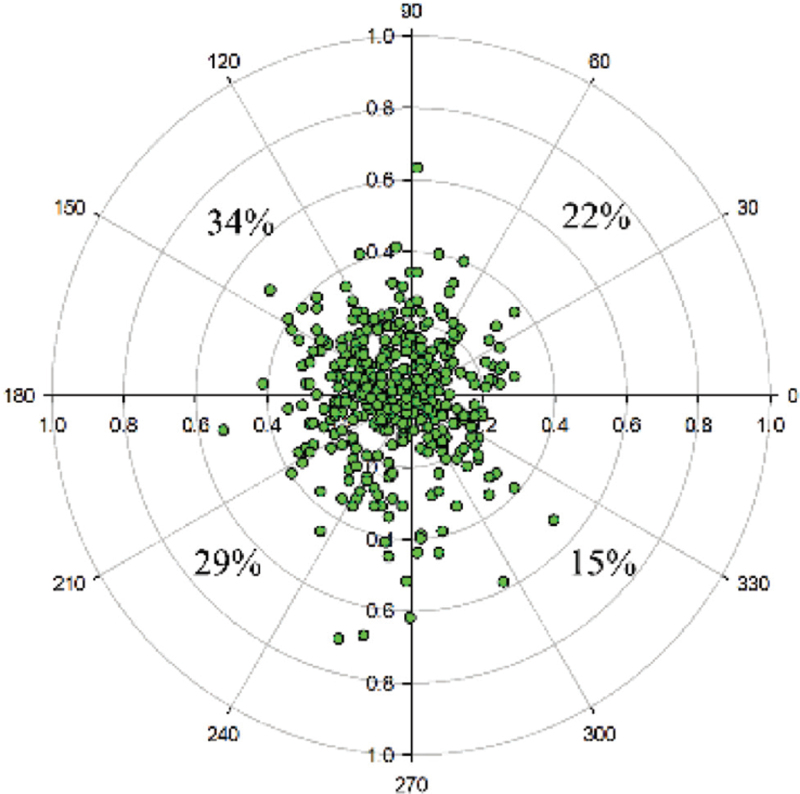
Location and distribution of coaxially sighted corneal reflex point suitable for corneal refractive surgery patients.

Figure 4 shows that the average distribution of *P-Dist* was 0.221 ± 0.098 mm (range: 0.008–0.526 mm); the *P-Dist* was 0.214 ± 0.092 mm in the right eyes and 0.228 ± 0.105 mm in the left eyes (*P* = .041). Figure 5 shows the distribution characteristics of the corneal quadrants of the coaxially sighted corneal light reflex point. The coaxially sighted corneal light reflex point was biased towards the upper temporal side of the corneal centre; 34% were above temporal, 29% were below temporal, 22% were above nasal and 15% were below nasal.

The ACD in the right eye was 3.53 ± 0.52 mm, and it was 3.58 ± 0.47 mm in left eye. There was no significant difference between the right and left eyes (*P* = .376). Table 2 shows the pupil size changes of the right and left eyes. There were no significant differences in pupil diameter between the right and left eyes under photopic conditions (*P* = .088). The mesopic pupil size was found to be lower in the right eyes than in the left eyes (*P* = .013). The pupil size changes (mesopic–photopic pupil diameter) were 2.79 ± 0.47 mm in right eyes and 2.81 ± 0.49 mm in left eyes. The pupil size changes in the left eyes were greater than they were in the right eyes (*P* = .030).

**Table 2:** Photopic and scotopic pupil size changes in the right and left eyes.

Category	Right eyes	Left eyes	Difference	*P*
Photopic pupil (mm)	3.28 ± 0.49	3.31 ± 0.51	0.03 ± 0.22	.088
Mesopic pupil (mm)	6.07 ± 0.72	6.12 ± 0.75	0.05 ± 0.33	.013
Change (mm)	2.79 ± 0.47	2.81 ± 0.49	0.03 ± 0.26	.030

Paired sample *t* test

**Figure 6:**
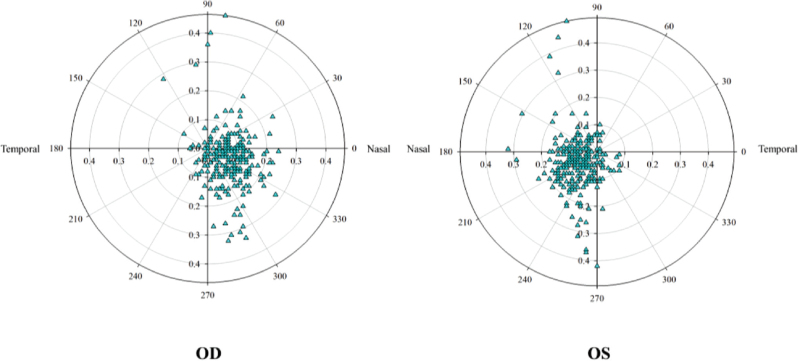
Vector diagram of pupillary centroid shift from scotopic to photopic condition in myopic patients (origin of coordinates is scotopic pupil center).

The dynamic change of pupil centre position of left and right eyes showed that the pupil centre position of left and right eyes under photopic condition showed no significant change in the *X*-axis and *Y*-axis directions (*P* > .05). Under scotopic conditions, the left eye *X*-axis was −0.046 ± 0.091 mm, the right eye *X*-axis was −0.152 ± 0.084 mm, with significant differences (*P* = .015), and the *Y*-axis direction showed no significant changes (*P* = .062). The measured centroid shift (scotopic-photopic) for the left eye as 0.127 ± 0.103 mm, and that of the right eye was 0.176 ± 0.139 mm, which was significantly different (*P *= .034) (Table 3). From scotopic to photopic condition, the pupil centroid shift of both eyes to the nasal inferior side, the nasal inferior shift was 88.69% in the right eye and 92.15% in the left eye (Fig. 6).

**Table 3:** Changes in horizontal and vertical axes of pupil centre under photopic and scotopic conditions.

	Right eyes	Left eyes	*t*	*P*
Photopic				
*X* Shift	−0.124 ± 0.104	−0.107 ± 0.132	1.068	.092
*Y* Shift	0.010 ± 0.142	−0.023 ± 0.137	0.869	.385
Scotopic				
*X* Shift	−0.152 ± 0.084	−0.046 ± 0.091	−2.448	.015
*Y* Shift	−0.014 ± 0.132	−0.032 ± 0.191	1.929	.062
Measured centroid shift(scotopic–photopic)	0.127 ± 0.103	0.176 ± 0.139	−0.647	.034

Paired sample *t* test.

**Figure 7:**
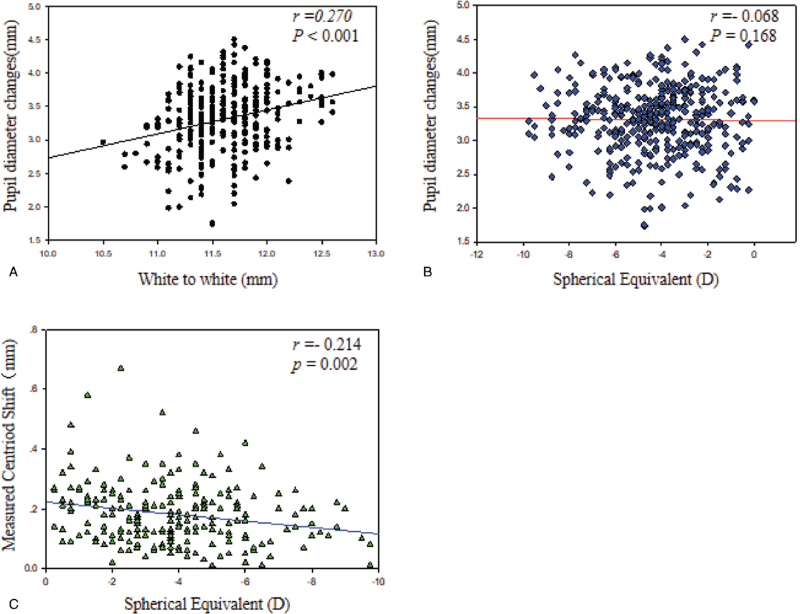
(A) Correlation between WTW and pupil diameter change (scotopic pupil diameter-photopic pupil diameter). (B) Correlation between SE and pupil diameter change (scotopic pupil diameter-photopic pupil diameter). (C) Correlation between SE and measured centroid shift.

**Figure 8:**
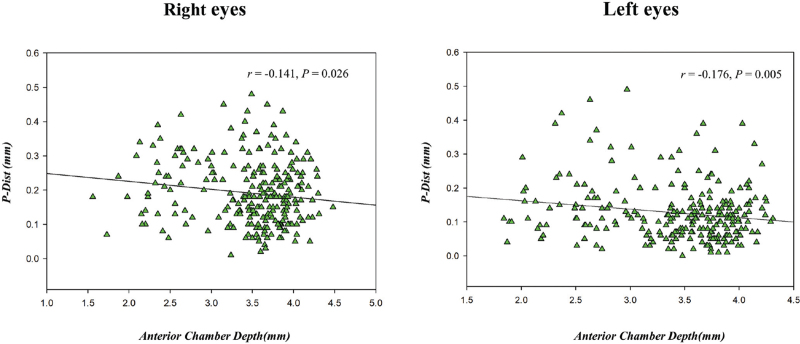
Relationship between ACD and *P-Dist* the right and left eyes (*r* = −0.141, *P* = .026 for the right eye; and *r* = −0.176, *P* = .005 for the left eye) *P-Dist* (distance from pupil centre to coaxially sighted corneal light reflex).

Figure 7A white to white (WTW) positively correlated with pupil diameter change (scotopic pupil diameter-photopic pupil diameter) (*r* = 0.270, *P* < .001). Figure 7B shows that equivalent spherical mirror (SE) had no correlation with pupil diameter change (*r* = −0.068, *P* = .168). Figure 7C shows that SE negatively correlated with measured centroid shift (the difference in distance to apex between the photopic and the mesopic pupil) (*r* = −0.214, *P* = .002). ACD negatively correlated with *P-Dist* for the right eye (*r* = −0.141, *P* = .026) and the left eye (*r *= −0.176, *P *= .005) (Fig. 8). There was no statistical difference in the higher-order aberrations between the right and left eyes in photopic conditions (*P* > .05), but in scotopic conditions, the coma in the right eye was 0.201 ± 0.143 μm, and that of the left eye was 0.255 ± 0.156 μm, with significant differences (*P* = .021)(Table 4).

**Table 4:** Comparison table of high-order aberrations in patients with intraocular myopia before operation.

	Photopic			Scotopic		
	Right eyes	Left eyes	*P*	Right eyes	Left eyes	*P*
HOA RMS	0.259 ± 0.122	0.253 ± 0.119	.411	0.387 ± 0.210	0.401 ± 0.209	.631
Coma	0.129 ± 0.113	0.133 ± 0.101	.092	0.201 ± 0.143	0.255 ± 0.156	.021
Spherical	0.117 ± 0.030	0.121 ± 0.037	.135	0.181 ± 0.126	0.207 ± 0.132	.077
Trefoil	0.143 ± 0.057	0.139 ± 0.048	.371	0.183 ± 0.118	0.195 ± 0.120	.183

Paired sample *t* test.

## 4 Discussion

Although angle kappa compensation combined with personalized femtosecond-assisted laser in situ keratomileusis has a good theoretical basis, there remains a significant gap between the actual and ideal visual quality.^[10]^ Factors influencing angle kappa include axial length, ACD and corneal curvature radius; however the proportion of influence from each factor has not been determined.^[11,12]^ Moreover, the size and central position of the pupil in corneal refractive surgery cannot be ignored. To ensure the accuracy of surgical positioning, it is necessary to ensure the matching with the preoperative pupil size and position, iris texture recognition, and corneal limbus recognition.^[13,14]^

Ablation centre point in corneal refractive surgery is controversial. Popular areas of research have focused primarily on *P-Dist*. Any change in lighting conditions, surgical stimulation and emotional tension would alter the pupil size, which shifts the pupil centre.^[15]^ Therefore, the pupil centre is not a desirable ablation centre point. The corneal intercept of the visual axis, which is defined as the line joining the fixation point to the fovea via the nodal point, has long been recommended as the ideal ablation centre. Nevertheless, it is difficult to locate this point precisely in practice.

At present, the target of non-interference pupil centre-corneal reflex line-of-sight tracking technology based on image processing is the pupil centre of the operated eye, and the direction of the visual axis is estimated by calculating the vector between the pupil centre and corneal reflex.^[16]^ The laser ablation centration is mostly centred on the coaxially sighted corneal light reflex point.^[17]^ If the positions of the two are different, the locked pupil centre needs to be adjusted accordingly.^[18]^ Therefore, according to the pupil size, centre position and dynamic change of angle kappa of the patients who are suitable for excimer laser surgery, obtained the individualized pupil centre displacement curve. During the operation, the pupil dynamics were monitored to adjust the angle kappa and calibrate the ablation centration in real time to ensure that each scanning-spot excimer laser was in the correct position.^[19]^

The angle kappa is not a fixed value, and it changes under different conditions.^[20]^ Influenced by factors such as intraoperative illumination, surgical stimulation, eye type, emotional tension, and accommodation convergence caused by close gaze indicator lights, there can be dynamic changes of pupil size and central position.^[21]^ Theoretically, when the size and the centre of pupil continuously change during surgery, the angle kappa also changes significantly.^[7,22]^

The results of this study indicate that the positions of the pupils’ centre of the left and right eyes were significantly different in under scotopic conditions. The pupil centers of the left eyes were basically distributed around the center of the cornea, and those of the right eye were 0.08 mm more temporal than those of the left eyes. In this study, coaxially sighted corneal light reflex is mainly distributed in the temporal side. Therefore, it is thought that the offset between the pupil center and the coaxially visible corneal light reflex of the left eyes is greater than that of the right eye. The present study indicates that ocular dominance occurred predominantly in the right eye (74.74%). We believe that hand dominance may affect which eye is held closer to the plane of a near task, especially when writing. Due to the inconsistent gaze target distance, the left eye needs more accommodation force to achieve the same state as the right eye (the right eye is closer), causing smaller pupils, shallower anterior chambers and a larger angle kappa relative to the right eye.

The relatively small degree of angle kappa in myopic eyes and the larger optical zone in myopic ablation compared to hyperopic ablation makes myopic ablation less sensitive to decentration. However, some studies suggest that ablation decentration of greater than 100 μm can induce aberrations in myopic treatments.^[2]^ In our study, more than 84.4% of eyes had *P-Dist* greater than 100 μm, 55.8% were greater than 200 μm, and 19.1% were greater than 300 μm.

The changes in the pupil centre positions of the left and right eyes suggest that the left eye should adjust a larger proportion of angle kappa when positioning in excimer laser surgery. Because the angle kappa offset of the left eye is greater than that of the right eye, to find the ablation centration point which is closest to the visual axis.^[23]^ This will avoid reduction of postoperative retinal imaging quality caused by unnecessary eccentric ablation.^[24]^ There was a negative correlation between ACD and *P-Dist*. Joon Hyung Yeo's^[25]^ study also showed that Angle Kappa decreased with the increase of ocular axis, and the longer ocular axis, the deeper anterior chamber. The longer the ACD, the smaller the Angle Kappa. The results are consistent with this study. In our study, measured centroid shift, when the light changed from scotopic to photopic, the pupil center shifted to the nasal side. Our results also showed a positive correlation between lateral WTW and pupil diameter change. Similar results have not been reported. This conclusion suggests that we should treat patients with large corneas during surgery to reduce the dynamic changes in pupil size with a low illumination environment as much as possible in order to improve the efficiency of pupil matching.^[26]^

The results of this study show that confirmed the importance of accurate location of ablation center and the necessity of reasonable adjustment of angle kappa vector ratio under different myopia degree and eye type. This study also has limitations: the insufficient samples and the individual variability may affect the results. The relationship between angle kappa compensation and visual quality need to be further studied. The digital relationship between various individualized ablation modes still needs further exploration.

## Acknowledgments

Thank my colleagues and teachers for their guidance, and my colleagues for helping me organize the data.

## Author contributions

**Data curation:** Wen-Qing Deng, Yingjun Li, Yu-Hui Fang, Yu-Jie Jia.

**Formal analysis:** Wen-Qing Deng.

**Investigation:** Xin-Yu Ru, Zheng-Ri Li.

**Methodology:** Wen-Qing Deng, Yingjun Li, Yu-Hui Fang.

**Project administration:** Shu-hua Lin, Yu-Hui Fang.

**Resources:** Shu-hua Lin, Xin-Yu Ru, Zheng-Ri Li.

**Software:** Wen-Qing Deng, Yu-Hui Fang.

**Writing – original draft:** Wen-Qing Deng, Wen-Qing Deng, Yu-Hui Fang.

**Writing – review & editing:** Yingjun Li.
